# Sex differences in off-target binding using tau positron emission tomography

**DOI:** 10.1016/j.nicl.2021.102708

**Published:** 2021-05-29

**Authors:** Ruben Smith, Olof Strandberg, Antoine Leuzy, Tobey J. Betthauser, Sterling C. Johnson, Joana B. Pereira, Oskar Hansson

**Affiliations:** a Clinical Memory Research Unit, Department of Clinical Sciences, Malmö, Lund University, Sweden; b Department of Neurology, Skåne University Hospital, Lund, Sweden; c Alzheimer’s Disease Research Center, Department of Medicine, Division of Geriatrics, University of Wisconsin, Madison, WI, USA; d Geriatric Research Education and Clinical Center, Wm. S. Middleton Memorial Veterans Hospital, Madison WI, USA; e Division of Clinical Geriatrics, Department of Neurobiology, Care Sciences and Society, Karolinska Institute, Stockholm, Sweden; f Memory Clinic, Skåne University Hospital, Malmö, Sweden

**Keywords:** Tau, PET, Off-target binding, Sex differences, Alzheimer’s disease

## Abstract

**Purpose:**

Off-target binding in the skull and meninges is observed in some subjects undergoing tau positron emission tomography (PET) and could potentially differ between men and women. In this study we elucidate sex differences in tau off-target binding using three different tau PET tracers.

**Methods:**

541 cognitively unimpaired amyloid-β negative participants underwent tau PET using [^18^F]flortaucipir (n = 165), [^18^F]RO948 (n = 189) and [^18^F]MK6240 (n = 187). Baseline SUVR-values were compared between females and males at the voxel level and using a region-of-interest (ROI) encompassing the skull/meninges. In addition, we assessed the cross-sectional relationship between baseline skull/meninges SUVR and age and assessed change in skull/meningeal SUVR values over time in a subsample with longitudinal data (n = 63).

**Results:**

Voxel-wise analysis showed higher meningeal off-target binding in women compared to men across all three tracers. The SUVRs in the skull/meningeal ROI were highest using [^18^F]RO948, followed by [^18^F]MK6240 and [^18^F]flortaucipir (p < 0.001). For all tracers, females showed higher skull/meningeal ROI retention (mean SUVR ± SD [^18^F]flortaucipir: 0.82 ± 0.14; [^18^F]RO948: 1.26 ± 0.30; [^18^F]MK6240: 1.09 ± 0.19) compared to men ([^18^F]flortaucipir: 0.70 ± 0.11; [^18^F]RO948: 1.10 ± 0.24; [^18^F]MK6240: 0.97 ± 0.17) (p < 0.001). For [^18^F]flortaucipir and [^18^F]RO948, off-target binding in the skull/meninges decreased with age.

**Conclusion:**

There is an effect of sex on off-target retention in the meninges/skull across [^18^F]flortaucipir, [^18^F]RO948, and [^18^F]MK6240 tau PET tracers.

## 1 Introduction

Several positron emission tomography (PET) radiotracers have been developed for detecting tau pathology in Alzheimer’s disease (AD) over the past decade (Leuzy et al., 2019). These include [^18^F]flortaucipir (Chien et al., 2013), and, more recently, [^18^F]RO948 (Honer et al., 2018) and [^18^F]MK6240 (Walji et al., 2016). Despite their specificity for tau aggregates, off-target retention in different regions can be seen using these compounds. Off-target binding has been most widely studied with the tracer [^18^F]flortaucipir, where it has been reported in the choroid plexus, (Baker et al., 2019, Lee et al., 2018, Pawlik et al., 2020) the basal ganglia (Baker et al., 2019, Choi et al., 2018, Smith et al., 2020) and binding to neuromelanin. (Hansen et al., 2016, Marquié et al., 2015) In studies using [^18^F]RO948, the off-target binding in the basal ganglia and choroid plexus is reduced compared to [^18^F]flortaucipir, but still present. (Smith et al., 2020) Off-target binding with [^18^F]MK6240 has been reported in the substantia nigra and meninges, but is not apparent in the basal ganglia or choroid plexus (Betthauser et al., 2019), and autoradiography suggests binding to neuromelanin. (Aguero et al., 2019) Off-target binding in the skull and meninges has not yet received much attention, but may complicate accurate signal quantification in cortical regions-of-interest (ROIs). By comparison to [^18^F]flortaucipir, off-target signal in the skull/meninges appears to be more pronounced in [^18^F]RO948 (Smith et al., 2020) and in [^18^F]MK6240 scans (Betthauser et al., 2019). There is increasing evidence showing that there are differences between women and men in tau PET retention (Smith et al., 2020, Wisch et al., 2020). However, to our knowledge no studies have addressed whether sex differences also affect off-target binding using tau PET. The aim of this study was therefore to assess whether off-target binding differed between men and women across [^18^F]flortaucipir, [^18^F]RO948, and [^18^F]MK6240. We used an imaging protocol adapted for and used in clinical studies (Buckley et al., 2019, Cho et al., 2019, Fleisher et al., 2020, Leuzy et al., 2020, Ossenkoppele et al., 2018, Pontecorvo et al., 2019, Sperling et al., 2019) in the form of static 20 min scans and standardized uptake value ratios (SUVR). In order to avoid the influence of true cortical (i.e., specific) binding on our off-target results, we included only amyloid-β (Aβ) negative cognitively unimpaired (CU) participants.

## 2 Methods

### 2.1 Participants

We included 541 Aβ-negative CU participants who underwent tau PET with either [^18^F]flortaucipir (BioFINDER-1 (Ossenkoppele et al., 2018), n = 27; Alzheimer’s Disease Neuroimaging Initiative (ADNI), n = 138); see Supplementary methods for PET details), [^18^F]RO948 (BioFINDER-2 (Leuzy et al., 2020), n = 189) or [^18^F]MK6240 (Wisconsin Registry for Alzheimer’s Prevention (WRAP) (Betthauser et al., 2019, Johnson et al., 2018), n = 187). For a small subset of participants, longitudinal data was also available ([^18^F]flortaucipir: n = 37, [^18^F]RO948: n = 26). All participants had no history of neurological or cognitive disorders and performed normally on cognitive tests. Aβ status (positive/negative) was determined using Aβ PET (BioFINDER-1: [^18^F]flutemetamol standardized uptake value ratio (SUVR), composite cerebellar, brain stem and white matter reference region; cut-off > 0.693; ADNI: [^18^F]florbetapir SUVR, whole cerebellum reference region; cut-off > 1.11; WRAP: [^11^C]PiB global distribution volume ratio > 1.2, whole cerebellum reference region) (Koscik et al., 2020) or the ratio of Aβ42 to Aβ40 in CSF (BioFINDER-2; Mesoscale Discovery Immunoassays, cut-off: <0.752). Written informed consent was obtained from all participants and local institutional review boards for human research ethics approved the studies at each site.

### 2.2 PET and MR imaging

Participants underwent tau PET imaging 80–100 min (BioFINDER-1) or 75–105 min (ADNI; these images were restricted to 80–100 min in our pipeline) after injection of 370 MBq [^18^F]flortaucipir (Smith et al., 2020); 70 –90 min after injection of 370 MBq [^18^F]RO948 (BioFINDER-2) (Leuzy et al., 2020) or 70–90 min after injection of 370 MBq [^18^F]MK6240 (Betthauser et al., 2019). High-resolution T1-weighted images were acquired on a 3 T Siemens MAGNETOM Skyra scanner (BioFINDER-1), 3 T Siemens MAGNETOM Prisma scanner (BioFINDER-2), 3 T Signa 750 (GE Healthcare; WRAP) and on various 3 T scanners in ADNI. These were used for image co-registration and template normalization. Images were non-linearly warped to template space via the ANTS based normalization of the anatomical scan to the MNI152 template. (Avants et al., 2014) PET images were motion-corrected, summed and co-registered to their corresponding T1-weighted MR images using an in-house developed pipeline (Leuzy et al., 2020). SUVR images were created using the inferior cerebellar cortex as the reference region. ROI-based measurements of off-target binding were performed in native space to avoid any potential bias from transformation of images into standard space. For the voxel-wise analyses, tau PET images were smoothed using a Gaussian kernel of 8 mm in SPM12 (Statistical Parametric Mapping software; https://www.fil.ion.ucl.ac.uk). All analyses were performed using non-partial volume error corrected data. For voxel-wise analysis a mask including the brain and meninges were applied to capture the off-target signal most relevant for the cerebral cortical binding.

### 2.3 Creation of the off-target skull/meningeal ROI

**Fig. 1:**
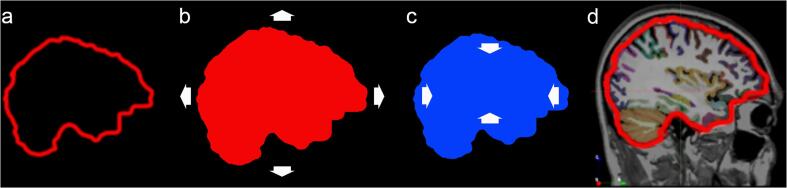
Generation of the skull/meningeal ROI. Schematic description of the generation of the skull/meningeal ROI. a) The FreeSurfer ROIs (Grey matter/white matter/CSF) were merged into one large volume of interest (VOI). b) The resulting VOI was dilated by 5 mm and all holes within the VOI were filled. c) The outer surface of the VOI was then eroded by 5 mm. The volume in c) (blue) was subtracted from the volume in b) (red) resulting in the VOI shown in d). The resulting VOI encompasses structures within 5 mm from the outer surface of the CSF layer surrounding the brain, sampling the meninges and inner parts of the skull. (For interpretation of the references to colour in this figure legend, the reader is referred to the web version of this article.)

The off-target skull/meningeal ROI used in this study has been described previously (Smith et al., 2020) and is described in Fig. 1. Briefly, the off-target mask was constructed using a series of morphological filters: first, the FreeSurfer grey matter, white matter and cerebrospinal fluid ROIs were merged into one volume and dilated by 5 mm. The dilated ROI was subjected to a fill-hole operation and subsequent erosion of 5 mm. Removing the resulting eroded voxels from the dilated mask yielded an exterior ROI encompassing a 5 mm border surrounding the surface of the brain that was used for estimating meningeal/skull binding. A 5 mm dilation was chosen since this represents the typical resolution of a PET-scanner, with the ROI therefore capturing off-target binding of potential relevance for the cerebral cortex ROIs.

### 2.4 Statistics

Analyses of variance with post hoc Tukey’s honest significant test (continuous variables) and chi-square tests (dichotomous variables) were used to assess differences in cohort demographics. Comparisons of skull/meningeal retention between men and women were performed using Student’s *t*-test. Correlations with age were carried out using Spearman correlations. All ROI-based statistical analyses were performed in R, version 3.6.2. Voxel-wise comparisons of tau PET images between men and women were carried out in SPM12, using age as a covariate and a brain mask including the meninges. The voxel-wise analyses were adjusted for multiple comparisons with family-wise error (FWE) rate corrections at p < 0.05.

## 3 Results

### 3.1 Participant characteristics

Participant demographics are presented in Table 1. [^18^F]MK6240 and [^18^F]RO948 participants were younger compared to the [^18^F]flortaucipir cohorts. [^18^F]RO948 participants were marginally younger than [^18^F]MK6240 participants, and there was a higher prevalence of female participants in the [^18^F]MK6240 cohort compared to the other cohorts. All participants were tau-negative in a large cortical composite region (Supplementary Fig. 1).

**Table 1:** Participant demographics.

	[^18^F]Flortaucipir	[^18^F]RO948	[^18^F]MK6240
**n**	165	189	187
**Sex (f/m) % female**	93/72 (56%)	110/79 (58%)	130/57 (70%) ^a,b^
**Age yr (mean ± SD)**	71.1 ± 5.94^c^	64.6 ± 12.54 ^d^	66.7 ± 6.53 ^e^

All participants were cognitively unimpaired and β-amyloid negative. F - female, m - male, SD – standard deviation. ^a^ * Flortaucipir vs MK6240. ^b^ * RO948 vs MK6240. ^c^ *** vs RO948 and MK6240. ^d^ *** vs Flortaucipir and * vs MK6240. ^e^ *** vs Flortaucipir and * vs RO948. * p < 0.05, *** p < 0.001.

### 3.2 Voxel-wise analysis

**Fig. 2:**
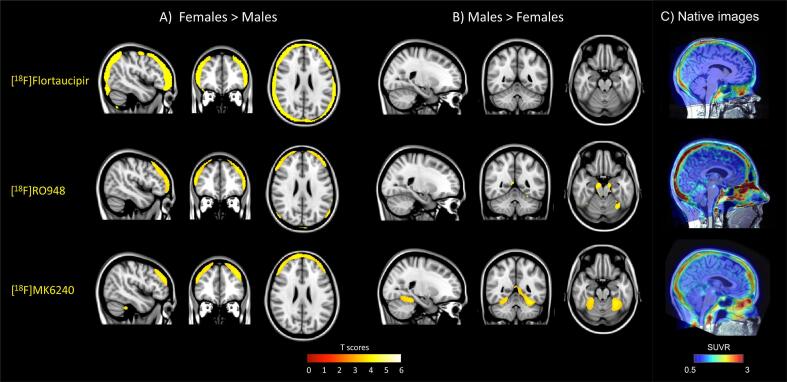
Voxel-wise comparison of binding between females and males. Voxel-wise comparison of tau PET retention using [^18^F]flortaucipir (upper row), [^18^F]RO948 (middle row), and [^18^F]MK6240 (bottom row) with a female > male contrast a) and a male > female contrast b). T-values are shown at the bottom. Results are corrected using FWE p < 0.05. c) Example SUVR images of participants with an increased skull/meningeal binding. SUVR – standardized uptake value ratio.

Tau PET signal in the skull/meninges was found to be significantly higher in females compared to males across all tau PET tracers (Fig. 2a). In contrast, males showed higher tau PET signal, by comparison to females, in small areas of the midbrain, superior parts of the cerebellum and brain stem using [^18^F]RO948 and in the superior parts of the cerebellum using [^18^F]MK6240 (Fig. 2b). No significant clusters were found in the males > females comparison using [^18^F]flortaucipir. Similar results were obtained after controlling for intracranial volume and baseline neocortical tau (Supplementary Fig. 2). Example images in native space for all three tracers are shown in Fig. 2c. As a control experiment, because of potential systematic differences in head size between males and females, we performed a similar analysis of [^18^F]flutemetamol PET scans within the BioFINDER-2 cohort (n = 144). We found no similar pattern of increased skull/meningeal retention in females in this comparison (Supplementary Fig. 3). Average SUVR-images in MNI space for males and females for all three tau tracers are shown in Supplementary Fig. 4.

### 3.3 Off-target binding in the skull/meninges

**Fig. 3:**
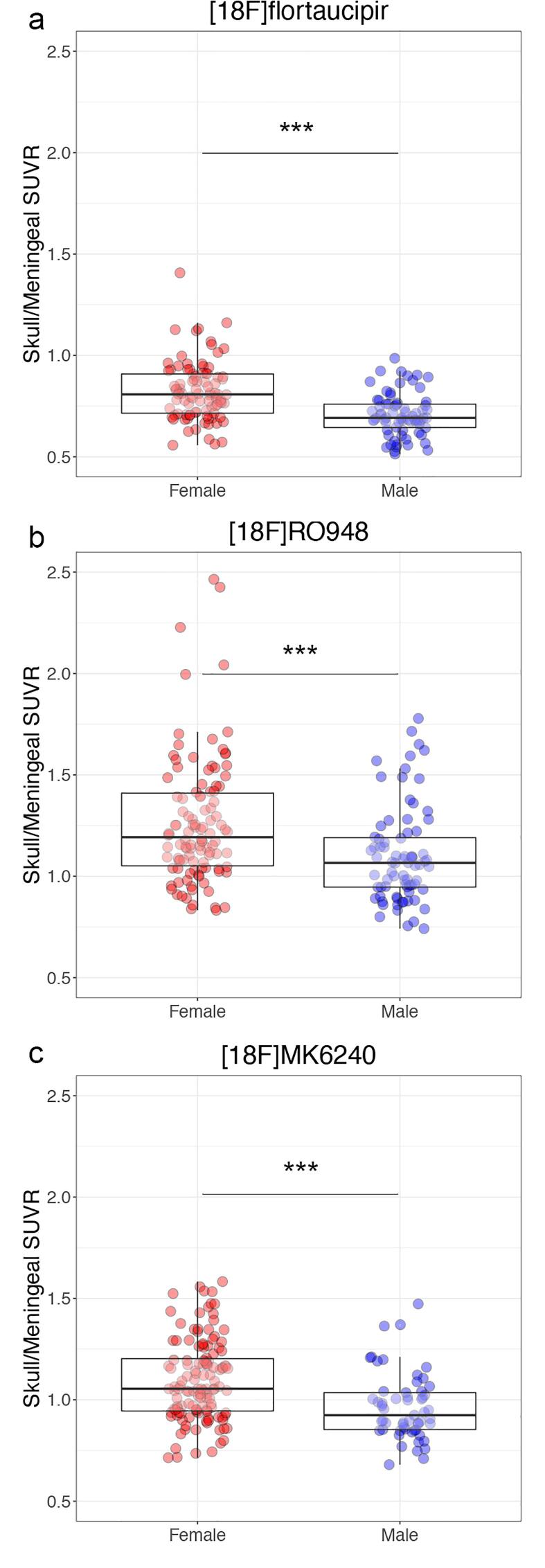
Sex differences in skull/meningeal off-target binding. SUVRs in the skull/meningeas ROI in females (red) and males (blue) using a) [^18^F]flortaucipir, b) [^18^F]RO948, and, c) [^18^F]MK6240. Boxes show median values and interquartile ranges with individual data points overlaid. *** = p < 0.001. (For interpretation of the references to colour in this figure legend, the reader is referred to the web version of this article.)

Retention in the skull/meninges ROI in native space was significantly higher in females compared to males using all three tau PET tracers (Fig. 3; [^18^F]flortaucipir, females vs males [mean SUVR ± SD]: 0.82 ± 0.14 vs 0.70 ± 0.11, p < 0.0001; [^18^F]RO948 1.26 ± 0.30 vs 1.10 ± 0.24, p < 0.0001; [^18^F]MK6240 1.10 ± 0.20 vs 0.96 ± 0.16, p < 0.0001). The SUVR values were lowest in the [^18^F]flortaucipir cohort, followed by the [^18^F]MK6240 and [^18^F]RO948 cohorts (all p < 0.001). We found a similar sex-difference regionally when analyzing the skull/meningeal retention overlying the frontal, parietal, temporal and occipital lobes separately in the BioFINDER-2 cohort (Supplementary Fig. 5).

**Fig. 4:**
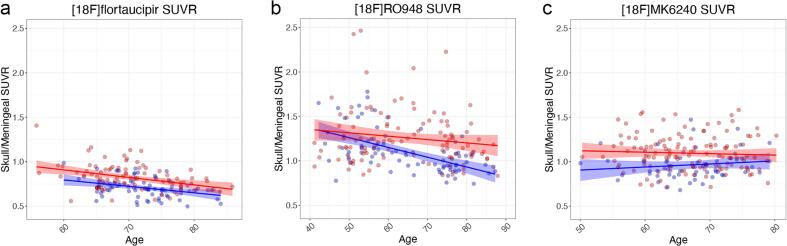
Correlations between age and skull/meningeal off-target binding in each sex. Skull/meningeal SUVRs plotted against age for a) [^18^F]flortaucipir, b) [^18^F]RO948, and, c) [^18^F]MK6240. Females are represented by red dots, males by blue dots. Lines are linear regressions and shaded areas correspond to 95% confidence intervals. (For interpretation of the references to colour in this figure legend, the reader is referred to the web version of this article.)

There was a significant negative correlation between age and the meningeal/skull SUVR of [^18^F]flortaucipir (Fig. 4; rho = −0.38, p < 0.0001) and [^18^F]RO948 (rho = −0.31, p < 0.0001), but not of [^18^F]MK6240 (rho = −0.02, p = 0.77). Using longitudinal [^18^F]flortaucipir and [^18^F]RO948 data, no sex differences were observed in the rate of change in meningeal/skull SUVRs per year (Supplementary Fig. 6; [^18^F]flortaucipir females vs males [mean ± SD]: −0.012 ± 0.05 vs 0.0002 ± 0.04, p = 0.21; [^18^F]RO948: 0.047 ± 0.099 vs 0.017 ± 0.071, p = 0.46). The variability in meningeal/skull SUVRs between baseline and follow-up scans (absolute percentage SUVR change, mean ± SD; female vs males) was 4.8%±4.5% vs 4.3%±3.1% for [^18^F]flortaucipir and 7.6%±7.1% vs 5.9%±5.4% for [^18^F]RO948. For comparison, absolute percentage change across the neocortex (mean ± SD; female vs males) for [^18^F]flortaucipir was 2.4%±3.5% vs 1.8%±1.5% and 2.4%±1.7% vs 1.7%±1.3% for [^18^F]RO948.

### 3.4 Correlation of off-target binding to disease and medication

In the BioFINDER-2 subsample we looked into available medical history and data on medication use as well as plasma C-Reactive Protein (CRP) levels. We did not find any correlation of the off-target signal to the use of anti-inflammatory medication, autoimmune inflammatory disease or levels of CRP (t-tests, p = 0.73, p = 0.21 and spearman correlation p = 0.94 respectively). Further, no associations were found with diabetes, hypertension, or the use of antihypertensive drugs or antidepressant medication. There were weak significant effects suggesting lower off-target retention in patients taking platelet inhibitors (p = 0.012) and lipid lowering (p = 0.017) medication, however, these did not survive correction for multiple comparisons.

## 4 Discussion

In this study, we showed that sex differences in skull/meningeal off-target binding are consistent across [^18^F]flortaucipir, [^18^F]RO948 and [^18^F]MK6240, three structurally different tau PET tracers. Skull/meningeal signal was more pronounced in the two more recently developed PET tracers ([^18^F]RO948 and [^18^F]MK6240) and was higher in [^18^F]RO948 compared to [^18^F]MK6240. The general off-target binding to the meninges/skull was low for [^18^F]flortaucipir, with mean values below the reference region SUVR value. For [^18^F]flortaucipir and [^18^F]RO948, we found a decrease in the intensity of the off-target binding with age. This was not observed using [^18^F]MK6240. In a small longitudinal sample of [^18^F]flortaucipir and [^18^F]RO948 scans we found a rather considerable variation in the off-target binding over time, with a mean absolute percent change per year of about 5% compared to 2% variation across the neocortex. However, no consistent between-sex differences in the longitudinal change were observed. The skull/meningeal off-target binding described in this report did not affect larger composite cortical ROIs such as the Braak imaging stage V-VI (Supplementary Fig. 1) and the magnitude of the off-target binding was lower in the [^18^F]flortaucipir scans. Nonetheless, the off-target binding may be of importance when studying ROIs close to the skull/meningeal such as the entorhinal, inferior frontal or occipital cortices. Moreover, our results stress the importance of balancing groups for sex or adding sex as a covariate in voxel-wise analyses.

The reason(s) underlying the increased skull/meninges binding in females compared to males are still unclear. The combination of sex differences with an off-target binding that decreases with age may suggest a role for sex hormones, although this remains speculative. A related potential explanation is hyperostosis frontalis interna, a benign, but rather rare, thickening of the inner side of the frontal bone of the skull that is found predominantly in women (She and Szakacs, 2004). The greater off-target binding we found near frontal areas using [^18^F]RO948 and [^18^F]MK6240 would support this hypothesis. A third possibility could be sex-related differences in metabolism of the radiotracers and an increased uptake of radiolabeled free fluorine in the skull in females. We found no significant relation of the increased skull/meningeal binding in females with inflammation, as measured by plasma CRP, to autoimmune inflammatory disease or to medication use. The areas that showed increased off-target binding in men compared to women in our study were limited to small regions in the upper parts of the cerebellum and brainstem, which have been previously reported as off-target regions. The reasons for the off-target binding in the superior cerebellum with [^18^F]RO948 and [^18^F]MK6240 in men compared to women remain elusive. The regions with increased retention in males were inconsistent between tracers and should be further replicated in future studies comparing the different tracers to determine whether these differences are driven by tracer properties or cohorts effects. It is important to note that the increased binding in females is not only a matter head sizes and of normalization to standard space since an increased retention of the radiotracers can also be seen in native space prior to normalization of the images as shown by the ROI-based results.

Our study is strengthened by the relatively large number of participants, which allowed us to find consistent results across tracers. There are a number of limitations of the current study. First, the PET and MRI images were acquired on different PET/MRI scanners and in cohorts that are not fully matched by age and sex. Though PET studies within the BioFINDER-1, BioFINDER-2 and WRAP studies were performed using the same scanner type, this was not the case for ADNI where different scanner types were used; as such, potential bias due to sex-differences across scanner types cannot be ruled out for the [^18^F]flortaucipir data. Second, our study did not use a head-to-head design and the comparisons of tau SUVRs between different tracers should be interpreted with this in mind. Third, we only have access to static scans and therefore differences in the kinetics of the binding of the off-target signal and true tau binding cannot be assessed in this dataset. Therefore, future dynamic studies will be needed to fully address the underlying causes of the sex-differences reported herein. The use of SUVRs for assessing binding in this off-target region is likely not ideal, but nonetheless, with the widespread use of SUVRs for analyzing images in clinical settings we find these results of large importance. Finally, our longitudinal sample was small, and conclusions made from these data should be considered preliminary pending replication in larger samples.

## 5 Conclusion

In conclusion, we found sex differences in the off-target binding of the meninges and skull using three different tau tracers, suggesting that balancing groups for sex in future treatment studies or controlling for sex in tau PET analyses may be advisable.

## 6 Ethics approval

The study procedures for each cohort have been approved by the local ethics review boards at the different participating sites.

## 7 Availability of data and material (data transparency)

Anonymized data will be shared by request from a qualified academic investigator for the sole purpose of replicating procedures and results presented in the article and as long as data transfer is in agreement with EU legislation on the general data protection regulation and decisions by the Ethical Review Board of Sweden and Region Skåne, which should be regulated in a material transfer agreement.

## 8 Authors' contributions

RS - data acquisition (BioFINDER1 and BioFINDER2), data analysis and drafting of manuscript; OS – data acquisition (BioFINDER1 and BioFINDER2), data processing and reviewing the manuscript; AL – reviewing the manuscript; TB – data acquisition, processing and QC (WRAP) and reviewing the manuscript; SCJ – data acquisition, funding (WRAP) and reviewing the manuscript; JBP – data analysis and drafting of manuscript; OH – data acquisition, funding (BioFINDER1 and BioFINDER2) and reviewing the manuscript.

## Funding

Work at Lund University was supported by the Swedish Research Council, the Knut and Alice Wallenberg foundation, the Swedish Alzheimer Foundation, the Swedish Brain Foundation, The Parkinson foundation of Sweden, the Marianne and Marcus Wallenberg foundation, the Strategic Research Area MultiPark (Multidisciplinary Research in Parkinson’s disease) at Lund University, The Parkinson Research Foundation, Region Skåne, the Swedish federal government under the ALF agreement, the Skåne University Hospital Foundation, and the Medical Faculty at Lund University.

The Wisconsin Registry for Alzheimer’s Prevention PET images used in this study were funded by NIH grants R01 AG027161 and R01 AG021155, and the Alzheimer’s Association (AARF-19–614533).

ADNI funding: Data collection and sharing for this project was funded by the Alzheimer's Disease Neuroimaging Initiative (ADNI) (National Institutes of Health Grant U01 AG024904) and DOD ADNI (Department of Defense award number W81XWH-12–2-0012). ADNI is funded by the National Institute on Aging, the National Institute of Biomedical Imaging and Bioengineering, and through generous contributions from the following: AbbVie, Alzheimer’s Association; Alzheimer’s Drug Discovery Foundation; Araclon Biotech; BioClinica, Inc.; Biogen; Bristol-Myers Squibb Company; CereSpir, Inc.; Cogstate; Eisai Inc.; Elan Pharmaceuticals, Inc.; Eli Lilly and Company; EuroImmun; F. Hoffmann-La Roche Ltd and its affiliated company Genentech, Inc.; Fujirebio; GE Healthcare; IXICO Ltd.; Janssen Alzheimer Immunotherapy Research & Development, LLC.; Johnson & Johnson Pharmaceutical Research & Development LLC.; Lumosity; Lundbeck; Merck & Co., Inc.; Meso Scale Diagnostics, LLC.; NeuroRx Research; Neurotrack Technologies; Novartis Pharmaceuticals Corporation; Pfizer Inc.; Piramal Imaging; Servier; Takeda Pharmaceutical Company; and Transition Therapeutics. The Canadian Institutes of Health Research is providing funds to support ADNI clinical sites in Canada. The grantee organization is the Northern California Institute for Research and Education, and the study is coordinated by the Alzheimer’s Therapeutic Research Institute at the University of Southern California. ADNI data are disseminated by the Laboratory for Neuro Imaging at the University of Southern California.

## Declaration of Competing Interest

RS, OS, AL, TJB, JBP report no disclosures. OH has acquired research support (for the institution) from AVID Radiopharmaceuticals, Biogen, Eli Lilly, Eisai, GE Healthcare, Pfizer, and Roche. In the past 2 years, he has received consultancy/speaker fees from AC Immune, Alzpath, Biogen, Cerveau and Roche. SCJ is a consultant for Roche Diagnostics and has received research support from Cerveau Technologies (neither are related to the work described here).
