# Supplementary material for: Sex differences in off-target binding using tau positron emission tomography
Source: Neuroimage Clin. 2021 May 29;31:102708. doi: 10.1016/j.nicl.2021.102708 (PMC8182304; doi:10.1016/j.nicl.2021.102708)
Supplement: Supplementary data 1 [file mmc1.docx]

*Supplementary information*

**Sex differences in “off-target” binding using tau positron emission tomography**

Ruben Smith, Olof Strandberg, Antoine Leuzy, Tobey Betthauser, for the Alzheimer’s Disease Neuroimaging Initiative, Sterling C. Johnson, Joana B. Pereira and Oskar Hansson.

***Supplementary Methods***

Data used in the preparation of this article were obtained from the Alzheimer’s Disease Neuroimaging Initiative (ADNI) database (adni.loni.usc.edu). The ADNI was launched in 2003 as a public-private partnership, led by Principal Investigator Michael W. Weiner, MD. The primary goal of ADNI has been to test whether serial magnetic resonance imaging (MRI), positron emission tomography (PET), other biological markers, and clinical and neuropsychological assessment can be combined to measure the progression of mild cognitive impairment (MCI) and early Alzheimer’s disease (AD). For up-to-date information, see www.adni-info.org.

***Supplementary Figures***

**Supplementary Figure 1. Neocortical retention of tau tracers**

**
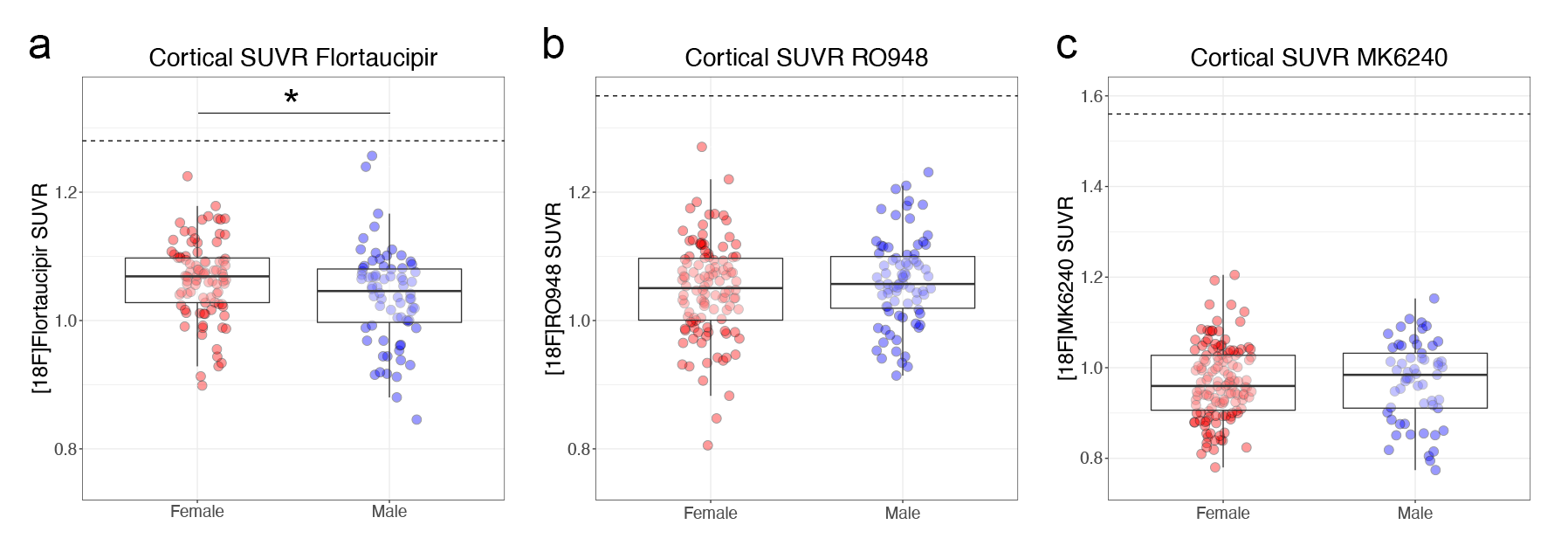
**

*Figure legend:* Cortical (Braak imaging stage V-VI) retention using a) [^18^F]Flortaucipir, b) [^18^F]RO948, and c) [^18^F]MK6240. Females are depicted in red (left) and males in blue (right). For [^18^F]Flortaucipir females had slightly higher SUVRs (1.06±0.06 vs 1.04±0.07, p=0.013), whereas there were no differences found in [^18^F]RO948 or [^18^F]MK6240 scans. Horizontal dashed lines indicate cut-off values for tau positivity established previously. [1-3]

**Supplementary Figure 2. Voxel-wise analysis correcting for intracranial volume and baseline neocortical tau values.**

**
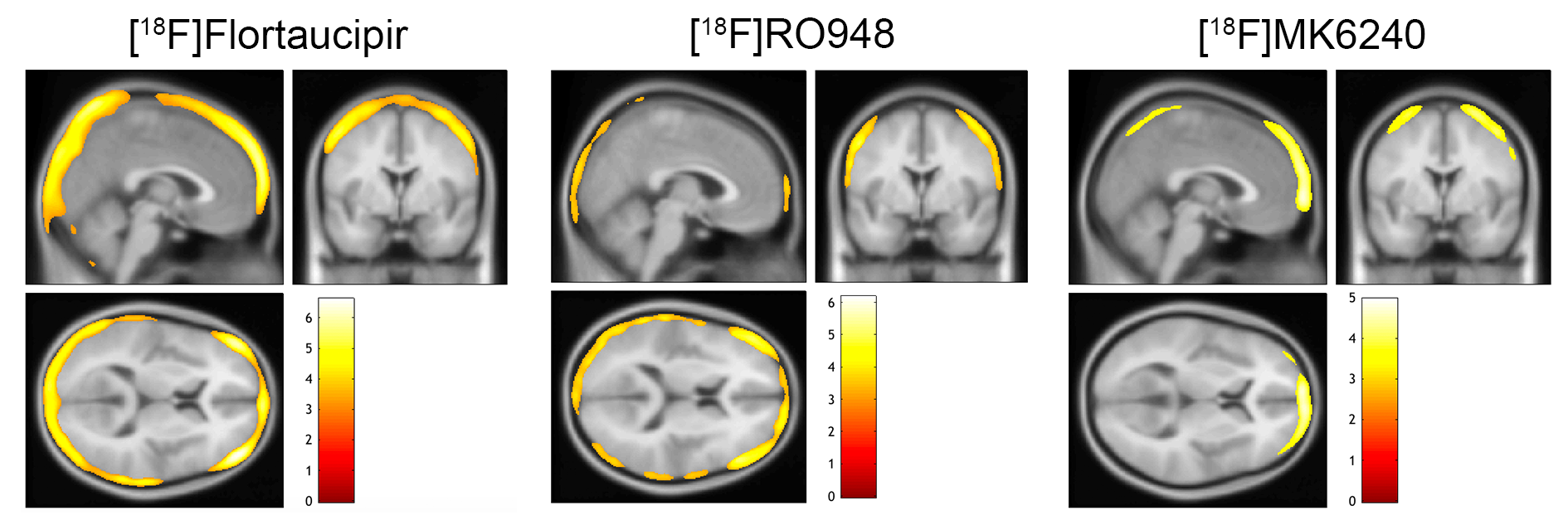
**

*Figure legend:* A similar analysis to the main manuscript Figure 2, but including intracranial volume and base-line neocortical tau as covariates. Significant voxels are FWE corrected at p<0.05.

**Supplementary Figure 3. Voxel-wise analysis of β-amyloid PET scans in BioFINDER-2**


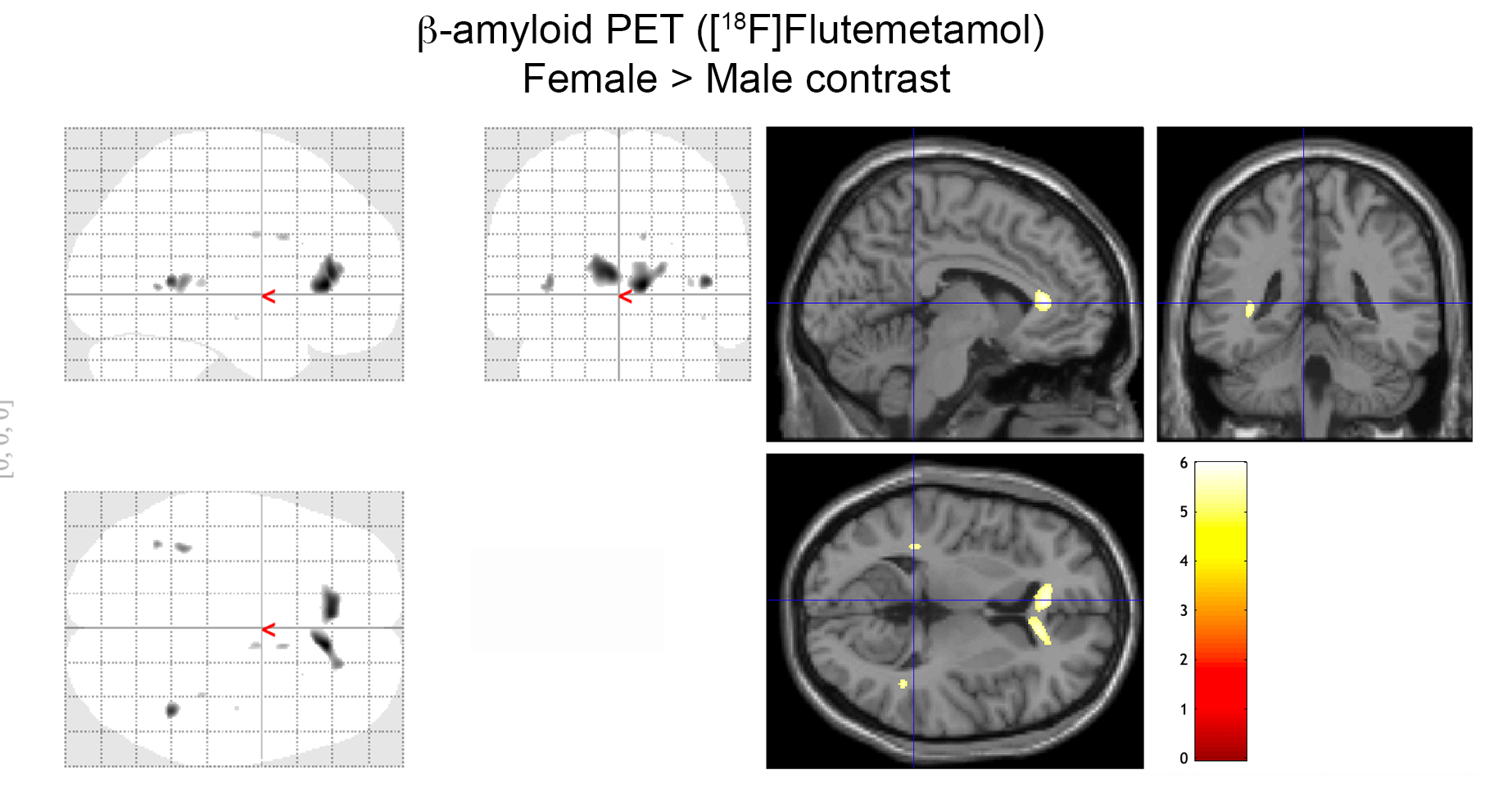


*Figure legend:* Voxel-wise analysis of 144 β-amyloid PET ([^18^F]flutemetamol PET) scans from the BioFINDER-2 cohort (males n = 65; females n = 79), masked for brain + meninges + skull. The left panel shows a glass brain of significant voxels (FWE corrected p <0.05). The right panel shows three sections overlaid on a single subject MRI (using spm 12). No effect of increased signal in the skull/meninges similar to the one seen using [^18^F]RO948 tau PET was seen using [^18^F]flutemetamol PET.

**Supplementary Figure 4. Average images of the three radiotracers in MNI-space for Males and Females.**

**
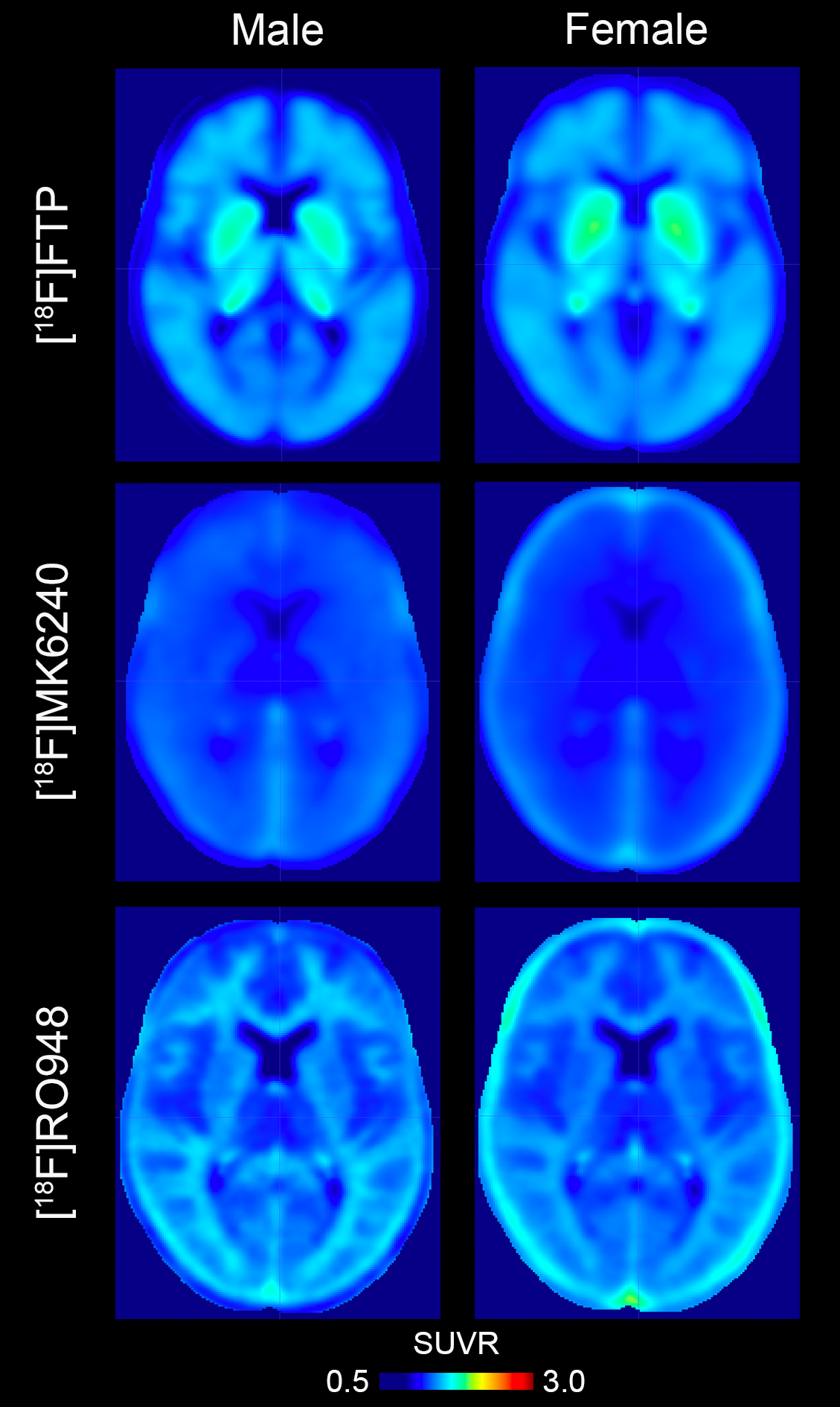
**

**Supplementary Figure 5. Regional off-target binding overlying the four main cerebral lobes.**

**
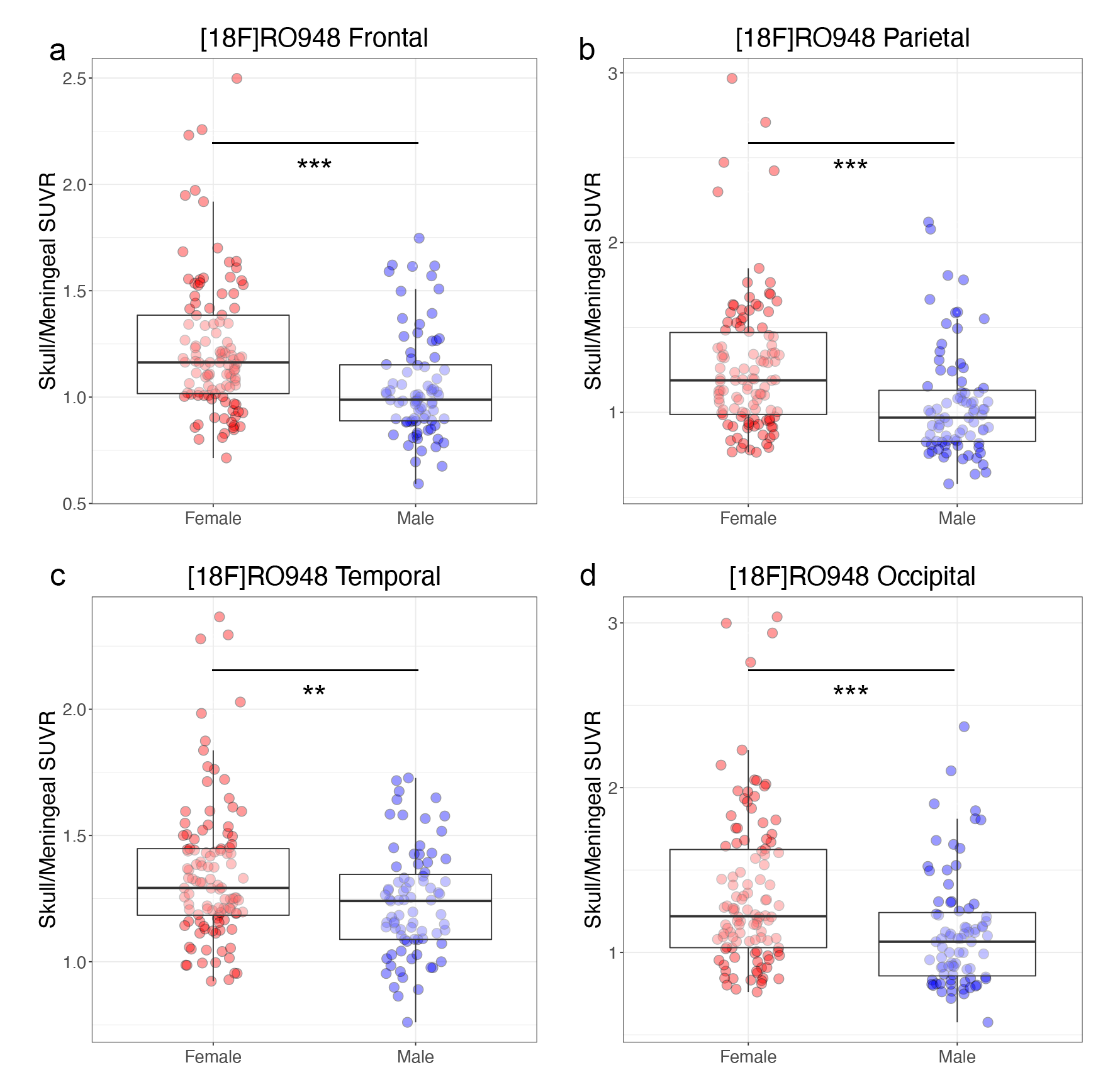
**

*Figure legend:* Regional off-target binding in the skull/meninges were assessed in ROIs overlying the four main cerebral lobes: a) frontal, b) parietal, c) temporal, and d) occipital. We find significant increases in females in all studied regions. ** p < 0.01; *** p < 0.001.

**Supplementary Figure 6. Longitudinal change in off-target retention**


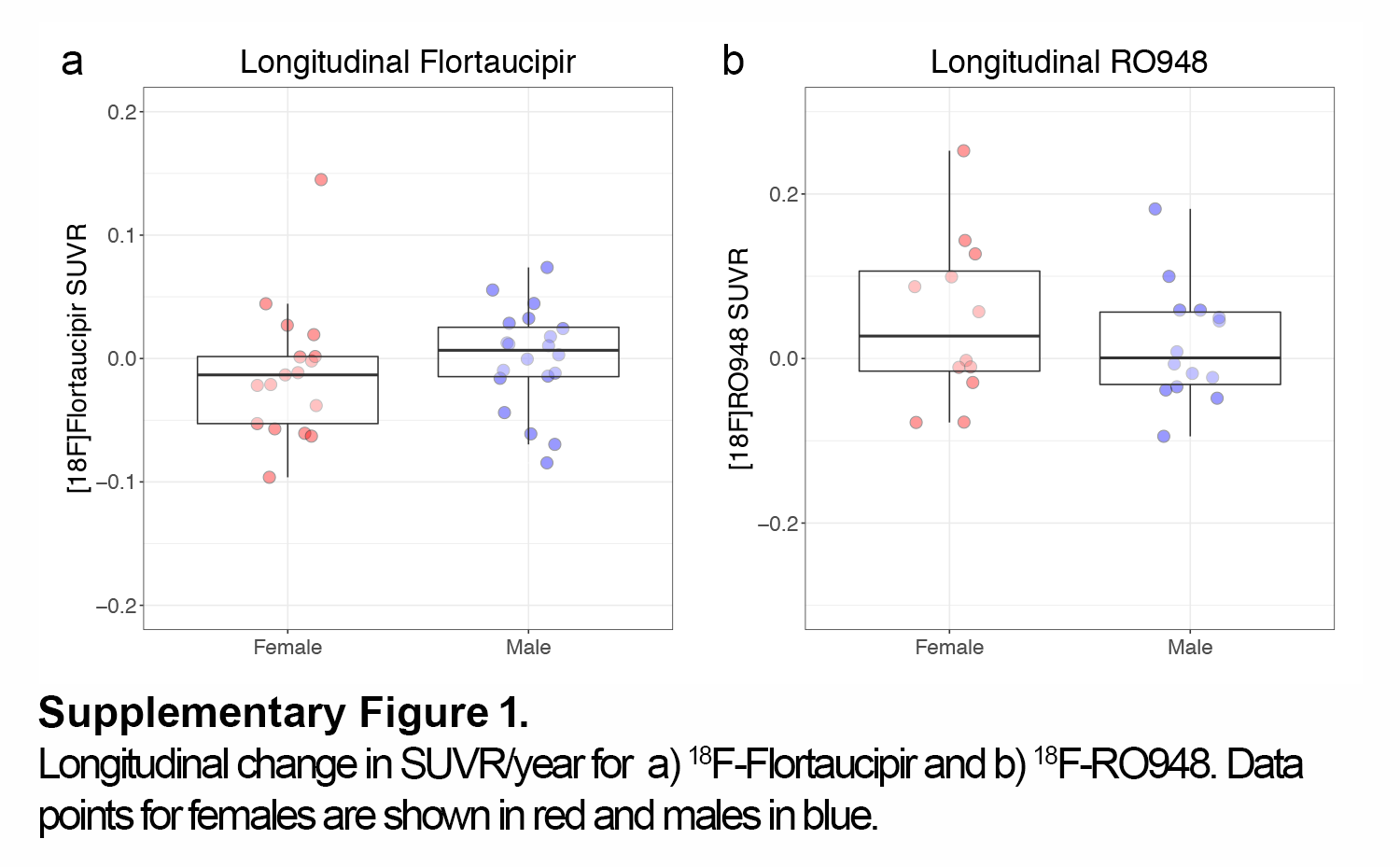


*Figure legend:* Longitudinal change in the meningeal off-target ROI shown in SUVR/year for a) ^18^F-Flortaucipir and b) ^18^F-RO948. Data points for females are shown in red and males in blue.

***Supplementary Table 1. Participant demographics in longitudinal sample***

|  | **[^18^F]Flortaucipir** | **[^18^F]RO948** |
| --- | --- | --- |
| **n** | 37 | 26 |
| **Sex (f/m) % female** | 17/20 (46%) | 12/14 (46%) |
| **Age yr (mean ± SD)** | 75.4 ± 5.60 ^a^ | 68.0 ± 11.2 ^a^ |

F - female, m - male, SD – standard deviation. ^a^ Flortaucipir vs RO948 p<0.01.

***References***

1. Leuzy A, Smith R, Ossenkoppele R, Santillo A, Borroni E, Klein G, et al. Diagnostic Performance of RO948 F 18 Tau Positron Emission Tomography in the Differentiation of Alzheimer Disease From Other Neurodegenerative Disorders. JAMA Neurol. 2020;77:955-65. doi:10.1001/jamaneurol.2020.0989.

2. Ossenkoppele R, Rabinovici GD, Smith R, Cho H, Scholl M, Strandberg O, et al. Discriminative Accuracy of [18F]flortaucipir Positron Emission Tomography for Alzheimer Disease vs Other Neurodegenerative Disorders. JAMA. 2018;320:1151-62. doi:10.1001/jama.2018.12917.

3. Pascoal TA, Therriault J, Benedet AL, Savard M, Lussier FZ, Chamoun M, et al. 18F-MK-6240 PET for early and late detection of neurofibrillary tangles. Brain. 2020;143:2818-30. doi:10.1093/brain/awaa180.
